# Recent advances in site-specific transgene insertion into the maize genome using recombinases and genome editing endonucleases

**DOI:** 10.3389/fpls.2025.1712585

**Published:** 2025-12-01

**Authors:** Marcos Fernando Basso, Maisa de Siqueira Pinto, Juliana Vieira Almeida Nonato, Ricardo Augusto Dante, Juliana Erika de Carvalho Teixeira Yassitepe

**Affiliations:** 1 Genomics for Climate Change Research Center, Universidade Estadual de Campinas, Campinas, Brazil; 2 Centro de Biologia Molecular e Engenharia Genética, Universidade Estadual de Campinas, Campinas, Brazil; 3 Embrapa Agricultura Digital, Barão Geraldo, Campinas, Brazil

**Keywords:** binary vector, homology-directed repair, transfer DNA, transgene insertion, prime editing

## Abstract

Random insertion of T-DNA into the maize genome requires the generation of many transgenic events, resulting in high cost and extensive development time. In contrast, site-specific transgene insertion (SSTI) in highly stable genomic regions emerges as an interesting and more viable alternative, as it allows obtaining elite lines in less time and effort. FLP/FRT and CRISPR/Cas9 homology-directed repair (HDR) strategies, as well as their combinations, are currently the most effective for SSTI in maize. The FLP/FRT system still depends on generating a high number of transgenic events, selecting a recombinant target line (RTL), and co-transforming this RTL with a second T-DNA, since there is no prior information on whether the insertion site is considered stable. In turn, CRISPR/Cas9 HDR requires prior information about the insertion site. From this principle, SSTI is effectively targeted by CRISPR/Cas9 HDR, requiring a smaller number of transgenic events. Furthermore, other strategies have been used for SSTI in animal cells and other plant species, and are very promising for establishment in maize as well. Herein, we highlight the importance of SSTI and the advances made in identifying genomic safe harbors in the maize genome. Furthermore, we emphasize the potential of the FLP/FRT system, CRISPR/Cas9 HDR, and CRISPR-associated recombinases and polymerases. We also offer insights into binary and ternary vector strategies, transgene delivery systems, maize tissue culture, and SSTI event genotyping. Finally, we highlight the importance of rigorous quality control for elite lines containing STTI. Therefore, this study provides insights and trends into SSTI in maize mediated by recombinases and genome editing endonucleases.

## 1 Introduction

Maize is cultivated globally, with a planted area of 90.6 million acres and a total production of 1,214,171 million metric tons for the 2024/25 harvest year. The United States, China, Brazil, and the European Union are the four largest producers, accounting for 31, 24, 10, and 5% of world production, respectively (USDA, 2025b). The United States, Brazil, and Argentina are the three largest exporters, while Mexico, the European Union, and Japan lead imports in the 2024/25 commercial year (USDA, 2025b). This production serves mainly as raw material for the production of animal protein, vegetable oil, and first-generation bioethanol, and to a lesser extent, for human food and industrial products (Kaur et al., 2025). Although projections suggest an increase in maize production through the 2034/35 harvest year, concerns remain about whether this will be sufficient to meet growing demand (USDA, 2025a). Global maize cultivation has faced challenges due to the effects of climate change, which results in exacerbated biotic and abiotic stresses, thus requiring the development of cultivars more adapted to cross-stress (Basso et al., 2024). Some necessary traits, such as resistance to specific insect pests, reduced susceptibility to certain abiotic stresses, and adaptation to nutritional limitations, are not available or cannot yet be achieved through traditional breeding (Singh et al., 2025; Basso et al., 2025b; Araújo-Lopes et al., 2025). Additionally, it is challenging to adapt tropical and temperate maize cultivars to different climatic conditions (Choquette et al., 2023; Basso et al., 2025a). To date, 307 maize transgenic events have been commercially developed by seven multinational companies, mainly used in combination to stack multiple traits in a given maize cultivar (Basso et al., 2024). These maize events overexpress genes encoding functional proteins, except for events MON-87411–9 and MON-94804, which overexpress, respectively, a dsRNA that effectively down-regulates the *DvSnf7* gene of *Diabrotica virgifera virgifera* (Head et al., 2017) and an artificial miRNA to down-regulate the *GA20ox-3* and *GA20ox-5* genes, resulting in maize plants with reduced stature (Paciorek et al., 2022). Most of these events carry transgenes encoding heterologous proteins and exhibit traits associated with resistance to herbicides and insect pests, while a smaller number are associated with drought tolerance, increased ear biomass, enhanced photosynthesis and yield, fertility recovery, phytase thermotolerance, brazzein protein, increased lysine production, and α-amylase thermostability (Basso et al., 2025c; ISAAA, 2025). These events were generated using classical transgenesis methods, which involved the random insertion of transfer DNA (T-DNA) into the maize genome and the rigorous selection of elite lines among hundreds of transgenic events. Therefore, the number of transgenic events generated for this purpose varies from a minimum required for proof-of-concept studies to a number that can reach a few hundred to a thousand when the goal is to develop elite transgenic lines with high potential to become commercial events (Basso et al., 2019, 2020; Yassitepe et al., 2021). However, the production, genotyping, and phenotyping of numerous transgenic maize events remain a significant challenge for biotechnology organizations worldwide (Pereira et al., 2025). The insertion of transgenes at random locations within the maize genome, whether mediated by *Agrobacterium tumefaciens*, biolistics, or the combination of these two delivery systems, makes this large-scale production process both inevitable and expensive (Basso et al., 2020). Random insertion produces more than 90% of transgenic events that often result in insertion at undesired positions and unstable transgene activity, further aggravated by a non-negligible number of multicopy and truncated insertions (Betts et al., 2019). The identification of a few transgenic events with significant potential among the hundreds produced requires five to fifteen years and depends on many robust molecular and phenotypic assays under greenhouse and open field conditions. These elite lines should also exhibit high and stable expression of their transcriptional units across generations in different plant tissues, regardless of normal or adverse conditions, including different stages of plant development and responses to biotic and abiotic stresses (Basso et al., 2020). Furthermore, current regulatory standards demand that elite transgenic lines feature a single and intact copy of the transgene inserted into a genomic position that does not disrupt adjacent endogenous genetic elements (Wang et al., 2025a). Therefore, it is increasingly important to identify or generate complex trait loci to facilitate trait stacking and reduce linkage drag in maize crosses (Gao et al., 2020b). Site-specific transgene insertion (SSTI) in highly stable regions of the maize genome emerges as a more viable alternative, as it allows obtaining elite lines in less time, with less effort and financial resources, and with greater precision and quality. However, we still need to improve our knowledge about the advantages and limitations of existing technologies for SSTI in maize.

In this study, we review the timeline of SSTI in maize and highlight advances in identifying genomic safe harbors in the maize genome, the potential of the FLP/FRT system compared to the Cre/LoxP system and other recombinases, and CRISPR/Cas9 HDR compared to other endonuclease-mediated genome editing systems and CRISPR/Cas9 variants. We also provide insights into binary and ternary vector strategies, transgene delivery systems, and tissue culture for genetic transformation of maize. Finally, we emphasize the need for rigorous quality control of SSTI-containing transgenic events. Therefore, this study offers insights and trends to expand critical knowledge about SSTI in maize, aiming to reduce costs and time for the development of elite lines featuring novel and multiple traits with commercial potential.

## 2 Brief timeline of SSTI in maize

SSTI in maize started a long time ago, using tools that were not as efficient and simple as those that exist today. The first insights began using recombination systems in maize protoplasts (Lyznik et al., 1993), in stable transgenic maize (Lyznik et al., 1996; Anand et al., 2019), evaluating different genome editing nucleases (Svitashev et al., 2015; Kumar et al., 2015), using CRISPR/Cas9 (Svitashev et al., 2016 and 2016; Barone et al., 2020; Peterson et al., 2021), and the combination of these recombinases and genome editing systems (Gao et al., 2020b). Therefore, SSTI in maize can be organized into three main technological generations. The first generation is based on the FLP/FRT and Cre/LoxP recombination systems, which involves the random insertion of a “receptor transgene” containing recombination sites for flippase (FRT sites) or Cre (LoxP sites) recombinases into the maize genome, acting as a “landing pad” for other transgenes linked to agronomic traits (Lyznik et al., 1993; Anand et al., 2019). Among hundreds of transgenic events generated, an elite transgenic or recombinant target line (RTL) is selected and genetically co-transformed so that a second transgene of interest is inserted through programmable recombination onto or into the recipient transgene that was previously inserted in the desired position in the maize genome (Anand et al., 2019). In summary, this first pathway is effective in SSTI, but has limiting characteristics, is expensive, and time-consuming in maize. The second generation relies on meganucleases, zinc finger nucleases (ZFNs), transcription activator-like effector nucleases (TALENs), and clustered regularly interspaced short palindromic repeats and CRISPR-associated protein 9 (CRISPR/Cas9) systems, which involve site-specific double-strand DNA break (DSB) directed by these endonucleases and SSTI directed by the plant’s own HDR mechanism (Svitashev et al., 2015; Kumar et al., 2015). Although all of these endonuclease systems can be used, they have some important limitations compared to the CRISPR/Cas9 system or its variants. To date, the most effective and easy-to-operate endonuclease system for SSTI in maize is CRISPR/Cas9 (Svitashev et al., 2015; Barone et al., 2020). CRISPR/Cas9 directs site-specific DSBs with target specificity determined by guide RNA (gRNA), while a co-delivered free donor DNA fragment serves as a template to repair this DNA cleavage through the HDR pathway (Sánchez-Rebato et al., 2024). The relative simplicity of this genome editing technology has allowed the SSTI in maize in a very short time and at a lower cost. However, this CRISPR/Cas9-mediated insertion strategy requires prior knowledge of specific positions in the maize genome considered stable hotspots for directing transgene insertion (Gao et al., 2020b; Peterson et al., 2021). With this prior knowledge of the desired position and experience in CRISPR/Cas9, genetic transformation, vector strategy, and molecular and phenotypic characterization of transgenic events, this demand becomes a fully viable challenge in maize (Long et al., 2024; Liu et al., 2024c; Pereira et al., 2025). The third generation of SSTI is still in the establishment phase in maize, but these technologies have already been shown to be quite promising in other systems, some already established in plants. Among these new technologies are iterative Cre-LoxP (Sun et al., 2025), TATSI (Liu et al., 2024b; Liang and Wang, 2024), EXPERT (Xiong et al., 2025), CASTs (Tou et al., 2023), RBKI (Zhou et al., 2025), DPET (Liu et al., 2024a), and CRISTTIN (Lin et al., 2025) systems, PrimeRoot (Sun et al., 2024) and dCas9-SSAP (Wang et al., 2022a) editors, and λ-red recombineering (Zhou et al., 2011; Thomason et al., 2023) and bridge RNAs (Hiraizumi et al., 2024; Durrant et al., 2024). Notably, many of these techniques applied in plants were first developed in animal, human, or microbial systems.

## 3 SSTI mediated by the FLP/FRT system

**Figure 1:**
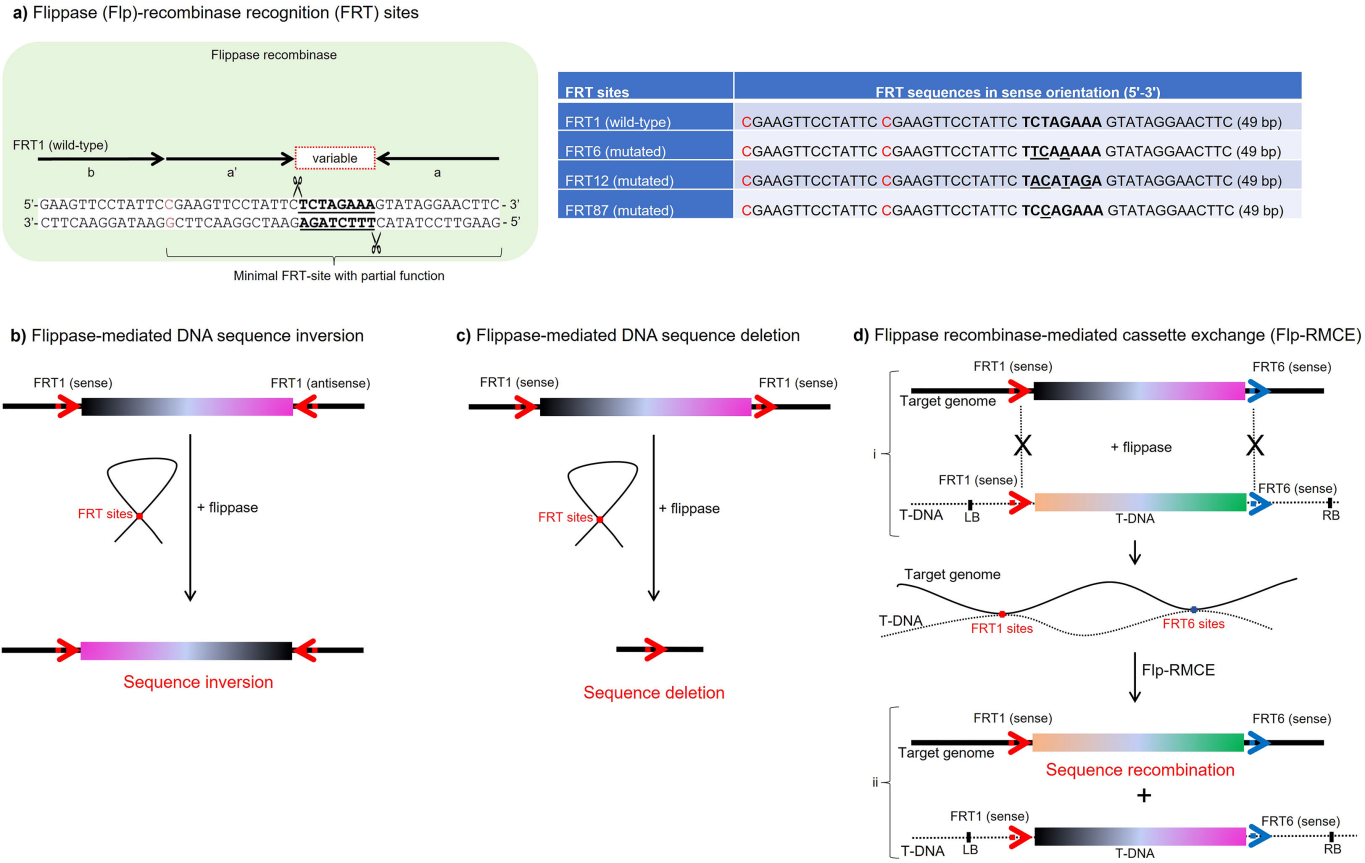
FLP/FRT recombination system. **(a)** Flippase (Flp)-recombinase recognition (FRT) sites commonly used for maize genetic engineering. FRT sites are 48 bp long, with a minimal of 34 bp, and an internal 8 bp that can be a little variable (bold letters). The internal 8 bp are flanked by 13 bp repeats identified by “b”, “a´”, and “a”, being “b” repeat behind an isolated base pair (red letter) (Turan et al., 2010). The internal 8 bp may have specific variations in bases (*e.g.*, FRT6, 12, and 87; underlined DNA bases), but still retain the ability to be recognized by the flippase. **(b)** Strategy for flippase-mediated DNA sequence inversion. DNA sequence (*e.g.*, as a transgene inserted into the maize genome by transgenesis) flanked by the same FRT sites (*e.g.*, FRT1 and FRT1), but in inverted orientations (sense and antisense), has the ability to form a loop or hairpin structure based on the sequence identity of the 48 bp FRT sites. Subsequently, the flippase enzyme encoded by the transgene itself or delivered independently recognizes this alignment of the FRT sites and mediates recombination (sequence inversion due to the planned orientation of the FRT sites). **(c)** Strategy for flippase-mediated DNA sequence deletion. Similarly, DNA sequence (*e.g.*, as a transgene inserted into the maize genome by transgenesis) flanked by the same FRT sites (*e.g.*, FRT1 and FRT1), in the same orientations (sense and sense), has the ability to form a loop or hairpin structure based on the sequence identity of the 48 bp FRT sites. Then, the flippase enzyme, encoded by the transgene itself or delivered independently, recognizes this alignment of the FRT sites and mediates recombination (sequence deletion due to the planned orientation of the FRT sites). **(d)** Strategy for flippase recombinase-mediated cassette or transgene exchange (Flp-RMCE). Similarly, the “recipient transgene” (*e.g.*, as a “landing pad” inserted into the maize genome by transgenesis) flanked by different FRT sites (*e.g.*, FRT1 and FRT6), in the same orientations (sense and sense), has the ability to align to the secondary transgene (donor transgene) also flanked by the same FRT sites (*e.g.*, FRT1 and FRT6 sites engineered in sense and sense orientations) based on the sequence identity of the 48 bp FRT sites. Then, the flippase enzyme encoded by the secondary transgene recognizes this alignment of FRT sites present in the landing pad and the secondary transgene and mediates recombination (sequence recombination due to the combination and orientation of the FRT sites used in the landing pad and secondary transgene). Finally, the donor transgene delivered into the secondary transgene is inserted into the landing pad, while the receptor transgene on the landing pad is inserted by recombination into the secondary transgene.

The recombinase superfamily is organized into tyrosine and serine groups, based on the active amino acid within the catalytic domain of these enzymes and distinct reaction mechanisms (Sales et al., 2025). Tyrosine recombinases, such as flippase derived from *Saccharomyces cerevisiae* and Cre derived from P1 bacteriophage, act in unidirectional or bidirectional manner between the two identical recognition sites (FRT and LoxP sites, respectively), while serine recombinases, such as phiC31 integrase derived from bacteriophage phiC31, act in unidirectional manner between two different recognition sites (AttP/attB sites) (Wang et al., 2011). The FLP/FRT system enables the precise inversion or deletion of a single DNA sequence, or allows precise recombination between two DNA sequences properly flanked by conserved FRT sites recognized by flippase (**Figure 1a**) (Schlake and Bode, 1994; Snaith et al., 1995; Turan et al., 2010). The orientation of these wild-type FRT pair sites, whether in the sense-antisense or sense-sense configuration, determines the flippase-mediated inversion or deletion of these DNA sequences, respectively (**Figures 1b, c**). Furthermore, combining heterologous FRT pair sites (wild-type and mutated) in sense-sense orientation facilitates effective flippase-mediated recombination and exchange between two distinct transgenes (flippase recombinase-mediated cassette or transgene exchange, Flp-RMCE), provided that the flippase is also supplied by one of these transgenes (**Figure 1d**). This system acts very similarly to Cre/LoxP, a well-known system based on Cre recombinase and LoxP sites, both used to produce marker-free transgenic maize (Du et al., 2019). In particular, the activity of the original flippase has an optimal temperature ranging from 20 to almost 30 °C, compared to Cre, which has an optimal temperature close to 37 °C (Buchholz et al., 1996). This difference in thermostability has led to the development of thermostable FLP variants for specific applications, such as FLPe (Buchholz et al., 1998). These optimal temperatures for maximum activity of these two recombinases are suitable for use in the genetic engineering of maize through *in vitro* tissue culture, which is maintained at a temperature around 25 °C. Although the Cre/LoxP system is as promising as the FLP/FRT system, it is already widely used to control the removal of morphogenic genes present in the T-DNA of binary vectors used in the genetic transformation of maize (Lowe et al., 2016; Wang et al., 2020; Aesaert et al., 2022). Furthermore, the Cre/LoxP system has not yet been satisfactorily optimized to achieve efficiency in removing these units containing morphogenic genes (Hernandes-Lopes et al., 2023). Therefore, its use for another important purpose and its low effectiveness place it in the background for SSTI in maize, leading to a preference for the FLP/FRT system (**Table 1**). In addition to these two well-established recombination systems, other recombinases have been preliminarily tested in maize, namely R (RS recognition sites), FLPe (FRT recognition sites), phiC31 integrase (attB/attP recognition sites), and phiC31 excisionase (attL/attR recognition sites), but further tests are still needed to determine their level of effectiveness in SSTI (Cody et al., 2020).

**Table 1:** Technical principles, advantages, limitations, applicable scenarios, operational complexity, and efficiency of FLP/FRT and CRISPR/Cas9 homology-directed repair (HDR) compared to other systems.

Characteristic	FLP/FRT system	Cre/LoxP system	CRISPR/Cas9 HDR	Meganucleases, ZFNs, and TALENs	TATSI	PrimeRoot editors	DPET	CRISTTIN
Require prior identification of genomic safe harbors	No	No	Yes	Yes	Yes	Yes	Yes	Yes
Require the generation of recombinant target lines	Yes	Yes	No	No	No	No	No	No
Sensitive to target DNA methylation	Unknown	Unknown	No	Yes	Unknown	Unknown	Unknown	No
Insertion of T-DNA or donor DNA	Mediated by flippase recombinase	Mediated by Cre recombinase	Mediated by the plant’s own DSB repair mechanism	Mediated by the plant’s own DSB repair mechanism	Mediated by transposase	Mediated by serine recombinase	Mediated by the plant’s own DSB repair mechanism	Mediated by the plant’s own DSB repair mechanism
Number of binary vectors for maize	A binary vector for transformation and another for co-transformation of the chosen recombinant target line	A binary vector for transformation and another for co-transformation of the chosen recombinant target line	A binary vector containing CRISPR/Cas9 and a second vector containing the donor DNA fragment	A binary vector containing CRISPR/Cas9 and a second vector containing the donor DNA fragment	A binary vector containing CRISPR/Cas9, transposase, gRNA, and the donor DNA fragment	A binary vector containing CRISPR/nCas9 fused with serine recombinase, pegRNAs, and the donor DNA fragment	A binary vector containing CRISPR/nCas9, MCP-Phi29, the first donor template fused to the MS2 aptamer, and a second vector containing the donor DNA fragment	A binary vector containing CRISPR/Cas9 fused with IMPα, gRNA, virD2, and a second binary vector containing the donor DNA fragment
Freedom-to-operate	Yes	Yes	CRISPR/Cas9 is linked to patents	ZFN and TALENs are linked to a patent claiming the *Fok*I cleavage half-domain	CRISPR/Cas9 variants are linked to patents	CRISPR/Cas9 variants are linked to patents	CRISPR/Cas9 variants are linked to patents	CRISPR/Cas9 variants are linked to patents
SSTI efficiency	Low	Very low	Low	Very low	Low	Low	Low	Low
Off-target effects	Low	Low	Low	Low	High	Low	Low	Low
Binary vector strategy and DNA delivery system	Highly complex	Highly complex	Highly complex	Highly complex	Complex	Highly complex	Highly complex	Highly complex
T-DNA or minimal expression cassette delivery	T4SS, viral vector, and biolistics	T4SS, viral vector, and biolistics	T4SS, viral vector, and biolistics	T4SS, viral vector, and biolistics	T4SS, viral vector, and biolistics	T4SS, viral vector, and biolistics	T4SS, viral vector, and biolistics	T4SS, viral vector, and biolistics
Minimum number of high-quality transgenic events generated	>100 recombinant target lines and 1–3 co-transformation events with precise SSTI	>100 recombinant target lines and 1–3 co-transformation events with precise SSTI	1–3 transformation events with precise SSTI	1–3 transformation events with precise SSTI	1–3 transformation events with precise SSTI	1–3 transformation events with precise SSTI	1–3 transformation events with precise SSTI	1–3 transformation events with precise SSTI
Insertion site for transgenes	Landing pad previously inserted into the recombinant target lines	Landing pad previously inserted into the recombinant target lines	Site-directed by the guide RNA and the homology arms of donor DNA fragment	Site-directed by the DNA-binding domain and the homology arms of donor DNA fragment	Site-directed by the guide RNA and insertion of donor DNA fragment mediated by NHEJ and/or transposase	Site-directed by the pegRNAs and insertion of the donor DNA fragment mediated by recombinase	Site-directed by the first donor template fused to the MS2 aptamer and insertion of the donor DNA fragment mediated by recombinase	Site-directed by the gRNA and insertion mediated by HDR of donor DNA anchored by the virD2 protein
Established for SSTI in maize	Yes	No	Yes	Yes	No	No	No	No

DSB, double-strand DNA break; ZFN, zinc finger nucleases; TALENs, transcription activator-like effector nucleases; T4SS, type IV secretion system; NHEJ, non-homologous end joining; pegRNAs, prime editing guide RNAs; SSTI, site-specific transgene insertion; DPET, DNA polymerase-mediated genome editing.

Primarily, the FLP/FRT system and FRT sites were used in engineered T-DNA sequences to flank and remove genetic elements that are essential only at the initial time point but undesirable at subsequent stages, such as in the generation of selectable marker-free transgenic maize (Srivastava and Ow, 2001, 2004). At the same time, it was also used for Flp-RMCE and gene conversion, reinforcing its potential for application in maize for SSTI (Lyznik et al., 1993, 1996; Djukanovic et al., 2006). To properly target Flp-RMCE, it is first necessary to generate RTLs containing an engineered transgene with heterologous FRT pair sites that function as landing pads for a second donor transgene, which also contains the corresponding heterologous FRT pair sites flanking transcriptional units associated with agronomic traits. These RTLs generated by random insertion represent high-quality transgenic events with desired site-specific insertion of these landing pads, which should be co-transformed with the second donor transgene that primarily includes the flippase and other genetic elements intended for insertion through recombination (Turan et al., 2010).

The first successful attempts at non-random insertion of transgenes linked to agronomic traits into the maize genome using recombinase were performed with the FLP/FRT system (Anand et al., 2019). Anand et al. (2019) demonstrated the successful development of maize RTLs with site-specific insertion of an engineered landing pad containing heterologous FRT pair sites. A stable RTL was subsequently co-transformed using a second T-DNA containing an engineered donor transgene for Flp-RMCE, resulting in efficiencies of 0.61 to 6.9% in Flp-RMCE frequencies. They highlighted that the transient expression of flippase may be sufficient for recovering Flp-RMCE events. The best results were obtained using the FRT1/FRT6 combination, and at least 2 to 3 rounds of transfer of these co-transformed explants during *in vitro* selection are necessary to detect some SSTI events. Furthermore, these authors also recommend considering strict quality control over these Flp-RMCE events, as several events have cassette exchange but may also contain additional random T-DNA insertions, backbone sequences, or other unintended DNA sequence insertions. Furthermore, some events may feature accurate recombination from the first FRT site (*e.g.*, FRT1 upstream of T-DNA or donor DNA) but illegitimate recombination at the second FRT site (*e.g.*, FRT6 downstream of T-DNA or donor DNA), and vice versa. The FLP/FRT system was also effective in the SSTI into rice (Nandy and Srivastava, 2011) and soybean (Li et al., 2009) RTLs, with satisfactory recombination efficiency of secondary transgenes. Similarly, the Cre/LoxP system was also effective for SSTI in rice, with similar efficiency to the FLT/FRT system (Nandy et al., 2015). Sun et al. (2025) obtained new LoxP sites with reduced reversibility and applied an artificial intellingence (AI)-assisted Cre recombinase engineering to generate an iterative Cre-LoxP system, which uses prime editing to increase editing precision, allowing scar-free insertions, deletions, replacements, inversions, and translocations at the chromosomal level. Meanwhile, sugarcane RTLs were generated containing a landing pad for subsequent transgene stacking mediated by the FLT/FRT and Cre/LoxP systems (Zhao et al., 2019). Finally, rearrangements of chromosome portions and construction of minichromosomes were also possible using these recombinases in different plant species, including maize (Qin et al., 1994; Yu et al., 2006, 2007; Yuan et al., 2017; Birchler et al., 2024).

Another homologous recombination system has been established in *Escherichia coli* for plasmid or genome editing, named λ-red recombineering (or λ-phage recombineering) system, and consists of the engineered bacteria (recombineering strain) that inducibly express the λ-red recombination genes encoding expressing the Exo, Beta, and Gam proteins of λ bacteriophage. Then, when transformed with a donor dsDNA fragment containing approximately 50 bp homology arms (engineered based on the target site for SSTI), Exo (exonuclease) generates overhangs into donor dsDNA, Beta binds to donor dsDNA and helps with recombination, while Gam prevents donor dsDNA degradation by nucleases (Thomason et al., 2023). The λ-red recombineering method was used to edit *Arabidopsis thaliana* genes cloned into an artificial chromosome library, and edited clones in binary vectors were transformed in *A. thaliana* by the floral dip method using *A. tumefaciens* (Murray and Gann, 2007; Zhou et al., 2011). Despite the effectiveness of editing the genes contained in the clones, the delivery of these edited DNAs to the plant generates several problems, such as transient expression, DNA fragmentation and partial degradation, off-target insertions, pleiotropic effects, and this method is limited to plant species with generated artificial chromosome libraries. Similarly, bridge RNAs are based on IS110 transposable elements identified in prokaryotes, which are elements flanked by conserved recombination sites, encoding an IS110 recombinase, and their self-release results in the formation of a circular dsDNA molecule containing an active promoter driving a non-coding RNA (ncRNA). This highly structured ncRNA is used for reinsertion into the genome by homologous recombination mediated by the heterocomplex IS110 recombinase, ncRNA, donor DNA sequence (transposon) flanked by recombination sites (Hiraizumi et al., 2024; Durrant et al., 2024). This system has been successfully established in bacteria. It is promising for plant systems, but its foundation, based on a transposon backbone, can produce several off-target insertions, especially in maize, which has many active transposable elements.

## 4 SSTI mediated by meganucleases, ZFNs, and TALENs

Unlike CRISPR/Cas9, whose Cas9 endonuclease is guided by the 20 bp gRNA to target the genome region based on sequence identity and protospacer adjacent motif (PAM) sequence, meganucleases, ZFNs, and TALENs target the DNA sequence through protein-DNA interactions (Dong and Ronald, 2021). Meganucleases are relatively small, highly specific, and rare-cutting endonucleases, originated from spliced introns or inteins, organized in at least five different families based on sequence identity and motifs, that tolerate some target site polymorphism but are sensitive to CpG methylation, composed of DNA-binding and double-stranded DNA cleavage domains at both N- and C-terminal (monomers), where each amino acid residue recognizes one DNA base, and their cleavage sites are typically 14 to 40 bp (**Table 1**) (Stoddard, 2011; Valton et al., 2012; Zaman et al., 2019). Therefore, these original meganucleases have well-defined DNA target specificity, whereas meganucleases engineered to have altered DNA binding specificity present few functional alternatives and often become inefficient, greatly limiting their use in SSTI (Lyznik et al., 2012; Daboussi et al., 2015). Nevertheless, they have been commonly used in their original or engineered form to remove selectable markers from T-DNA designed with these transgenes flanked by meganuclease recognition sites (Antunes et al., 2012; Yau and Stewart, 2013) and induce site-specific mutations (Gao et al., 2010; Djukanovic et al., 2013; Cigan et al., 2017). Furthermore, these meganucleases were used for SSTI or trait stacking in non-plant (Resly et al., 2025) plant systems, such as tobacco (Tzfira et al., 2003), cotton (D'Halluin et al., 2013), maize (D'Halluin et al., 2008; Ayar et al., 2013), *A. thaliana* (Fauser et al., 2012), tomato, and oilseed rape (Danilo et al., 2022). Therefore, it is clear that the biggest challenge in using meganucleases is engineering their activity to a specific DNA sequence and ensuring their high efficiency in cleaving double-stranded DNA. Meanwhile, the repair efficiency by the NHEJ or HDR pathways, with site-specific mutation or transgene insertion in maize, is comparable to that obtained with other endonucleases.

ZFNs are chimeric and relatively small endonucleases composed of an *in tandem* fusion of several zinc finger domains at the N-terminal with the catalytic domain of the *Fok*I endonuclease at the C-terminal, where each zinc finger domain is approximately 30 amino acids long and recognizes three DNA bases (Tovkach et al., 2010; Isalan, 2012). Its cleavage target site is typically 24 to 36 bp, considering the obligatory formation of ZFN dimers that recognize sense and antisense DNA strands, while the *Fok*I heterodimer directs the DSB (Negi et al., 2023). Although ZFNs are easier to engineer than meganucleases, they need to be designed to form two monomers, can be sensitive to CpG methylation, and are more likely to produce off-target effects (**Table 1**) (Katayama and Yamamoto, 2025). It has been shown that the catalytic domain of *Fok*I can be altered by other endonuclease domains, such as the ND1 endonuclease (Katayama and Yamamoto, 2025). Furthermore, ZFNs have been successfully used in different plant species to target site-specific mutations (Lloyd et al., 2005; Osakabe et al., 2010; Zhang et al., 2010; Curtin et al., 2011; Hilioti et al., 2016), insertion or exchange of transgenes in landing pads (Wright et al., 2005; Petolino et al., 2010; Cantos et al., 2014; Schiermeyer et al., 2019), and non-synonymous mutations in protein-coding sequences based on HDR using donor DNA template (Townsend et al., 2009; Ran et al., 2018). Particularly in maize, ZFNs were used for transgene insertion into landing pads (Ainley et al., 2013), gene editing (Shukla et al., 2009), and site-specific transgene stacking (Kumar et al., 2015), with an efficiency of approximately 1-5%. Therefore, although ZFNs are effective for maize genome editing, they have also been little used in SSTI.

In turn, TALENs are also large artificial endonucleases, composed of several *in tandem* fusions of 15 to 20 DNA-binding modules and a catalytic domain of the *Fok*I endonuclease (Osakabe and Osakabe, 2015). Each of these binding modules, of approximately 34 amino acids, has two repeating variable residues (RVD) that determine the binding specificity of this module to each DNA base. Each module recognizes 1 bp of one of the DNA strands, resulting in a target site of 30 to 40 bp, considering both sense and antisense DNA strands (Streubel et al., 2012). Therefore, TALENs are less complex to engineer, more efficient and specific, and with fewer off-target effects compared to meganucleases and ZFNs (Zhang et al., 2013). However, TALENs are sensitive to CpG methylation, more time-consuming, cost-ineffective, and have lower editing efficiency compared to CRISPR/Cas9 (**Table 1**) (Gaj et al., 2013). Furthermore, TALENs were used in several plant species to introduce site-specific mutations and gene knockout (Li et al., 2012; Christian et al., 2013; Sun et al., 2013; Lor et al., 2014; Wang et al., 2015; Demorest et al., 2016; Clasen et al., 2016; Luo et al., 2019; Qin et al., 2021), and SSTI (Čermák et al., 2015; Forsyth et al., 2016). Particularly in maize, TALENs have been used for targeted mutagenesis with similar efficiency to CRISPR/Cas9 (Liang et al., 2014; Char et al., 2015; Kelliher et al., 2017). Therefore, several advances were made with all three groups of endonucleases for site-directed mutation and SSTI, but the greatest number of successful genome editing efforts were achieved with CRISPR/Cas9 (Zhang et al., 2018). Despite the complexity and high cost of engineering these endonucleases, recent technological advances have allowed the synthesis of long DNA fragments at affordable prices. Finally, considering the SSTI in genomic safe harbors, which has a window of approximately 50 kbp, may facilitate the design of these endonucleases.

## 5 SSTI mediated by CRISPR/Cas9 HDR

**Figure 2:**
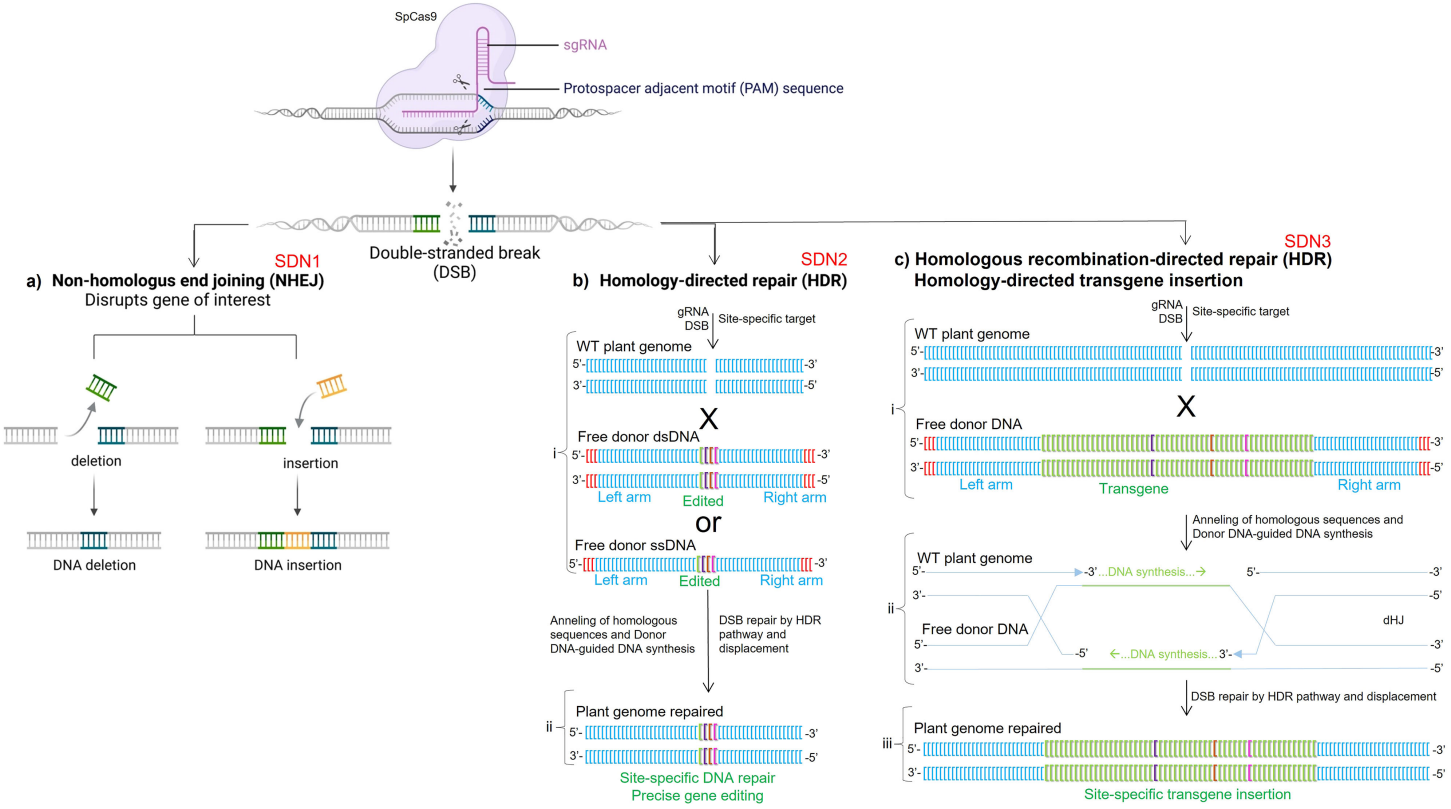
CRISPR/Cas9 technology for site-specific transgene insertion (SSTI) mediated by CRISPR/Cas9 homology-directed repair (HDR). **(a)** CRISPR/Cas9 non-homologous end joining (NHEJ) is used for insertion or deletion (*indels*) of DNA bases in non-coding or coding-protein sequences based on single guide RNA and Cas9, or deletion of larger DNA fragments based on two guide RNAs and Cas9. This double-strand DNA break (DSB) is repaired by classic non-homologous end joining (cNHEJ), since Cas9 generates blunt ends after cleavage (Xue and Greene, 2021). However, if sticky ends are generated, such as by meganucleases, ZFNs, TALENs, and Cas12a (Cpf1), DNA repair is also by microhomology-mediated end joining (MMEJ), short or long-range end resection, or single-strand annealing (SSA) (Xue and Greene, 2021). **(b)** CRISPR/Cas9 HDR is used to repair or insert single and multiple, site-specific, synonymous or non-synonymous substitutions in non-coding or coding-protein DNA sequences. A free donor DNA fragment with right and left arms is delivered into the target cell as double-stranded (dsDNA) or single-stranded (ssDNA) and used as a template for DNA repair. This DSB is repaired by non-crossover synthesis-dependent DNA strand annealing (Xue and Greene, 2021). **(c)** CRISPR/Cas9 HDR strategy used to SSTI into desirable genomic regions through HDR. A free donor DNA fragment or a desirable transgene, flanked by right and left arms, is delivered as double-stranded (dsDNA) and used as a template for HDR. In turn, the homology arms contribute to the transgene being erroneously recognized as part of the edited genome and therefore inserted by double Holliday junction (dHJ) during DNA repair (Bzymek et al., 2010; Bizard and Hickson, 2014). Site-directed nucleases (SDN) 1 to 3 are technical biosafety terms used to differentiate the types of alterations made through CRISPR/Cas9-mediated genome editing. sgRNA, guide RNA; WT, wild-type.

**Figure 3:**
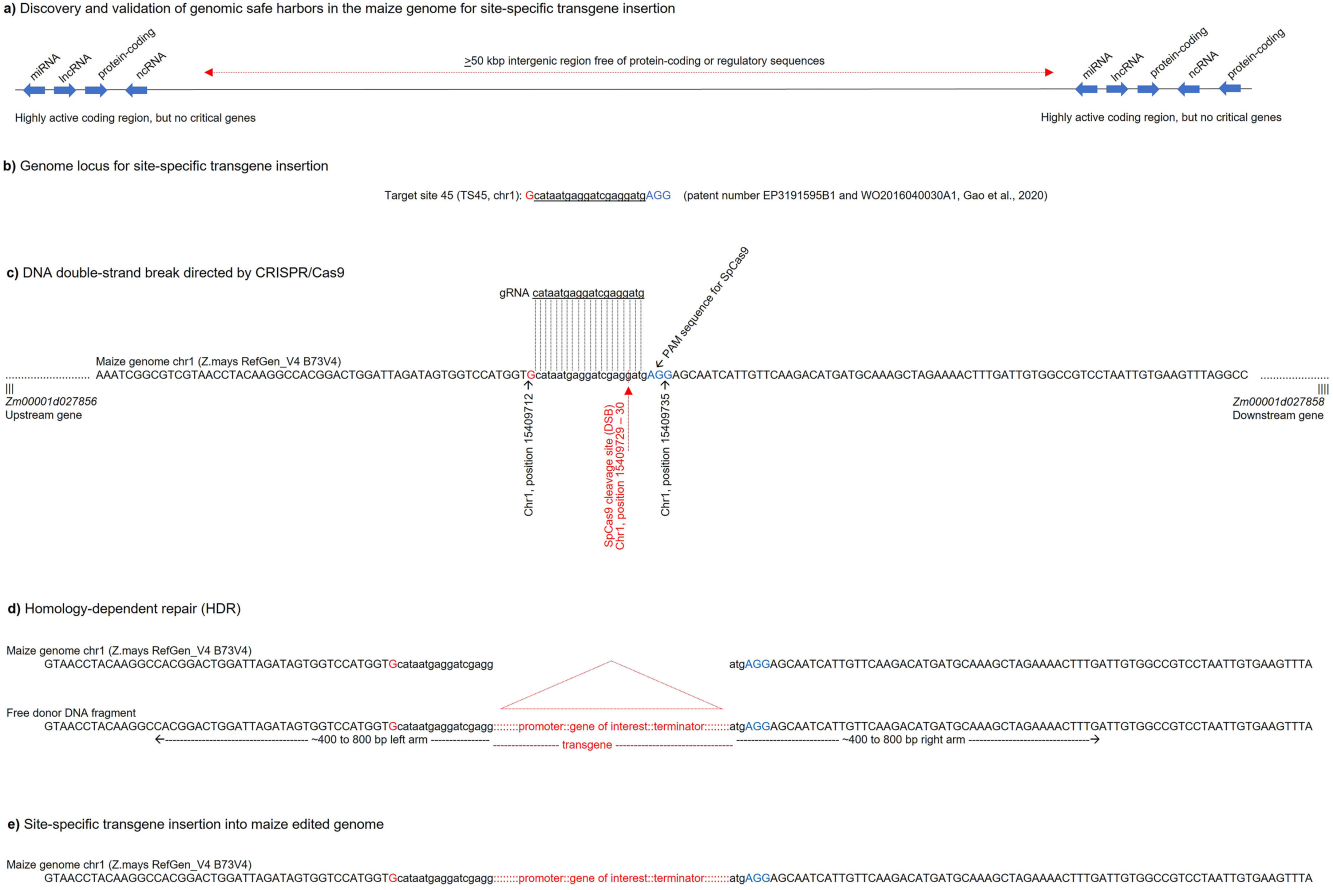
Genomic safe harbors and site-specific transgene insertion (SSTI) in the maize genome mediated by CRISPR/Cas9 homology-directed repair (HDR). **(a)** Discovery of genomic safe harbors in the maize genome based on large intergenic regions (>50 kbp) flanked by non-critical but highly active coding sequences. For example, genomic safe harbors with neighboring genes that exhibit stable and high expression across plant generations, in different organs, and under diverse growing conditions. **(b)** An example of a genomic safe harbor, TS45, was identified by Gao et al. (2020b) and experimentally validated as a stable position for SSTI in the maize genome, chromosome 1 (Chr1). **(c)** Double-strand DNA break (DSB) directed by CRISPR/Cas9 at TS45 position. Site-specific DNA cleavage by Cas9 is mediated by the specificity of gRNA and protospacer adjacent motif (PAM). **(d)** HDR of the DSB at position TS45 mediated by free donor DNA containing a desirable transgene flanked by right and left arms of ~400–800 bp. **(e)** SSTI at position TS45 mediated by the HDR pathway, especially by the homology arms flanking the donor DNA fragment and the plant DNA repair mechanism.

CRISPR/Cas9 HDR has been successfully used in maize to repair endogenous genetic elements to improve agronomic traits, such as genes associated with plant susceptibility to herbicides (Svitashev et al., 2015; Kaul et al., 2024) and transcriptional regulatory elements (Shi et al., 2017). From these advances, CRISPR/Cas9 HDR-mediated SSTI has been established in maize as a highly viable alternative that may require less time, financial resources, and effort to achieve elite lines compared to the FLP/FRT and Cre/LoxP systems (**Table 1**) (Barone et al., 2020). However, prior knowledge of the genomic safe harbors is a fundamental requirement for SSTI mediated by CRISPR/Cas9 HDR (Gao et al., 2020b). Ensuring that previously identified genomic safe harbors provide high transgenerational stability and activity to transgenes, the Cas9 targeting by gRNA and site-specific DNA cleavage are already well established in maize genetic engineering (Gao et al., 2020a; Peterson et al., 2021). In particular, a free donor DNA containing right and left arms of approximately 400 to 800, designed to flank the desired insertion site, provided by T-DNA or biolistics, will act in the HDR pathway (**Figure 2**). Thus, Cas9 will cleave the double-stranded DNA precisely at the desired position, such as within genomic safe harbors (**Figure 3a**), while free donor DNA containing the transgene(s) of interest will anneal to the target genomic DNA at this same position and serve as a repair template for the cleaved DNA, resulting in the insertion of the transgene mediated by the HDR pathway (**Figures 3b-e**) (Basso et al., 2020; Sánchez-Rebato et al., 2024).

One of the first SSTIs in maize using CRISPR/Cas9 HDR was achieved at the *LIG1* locus, specifically at the 5’-UTR of the *liguleless1* gene (Svitashev et al., 2015). These authors showed that by providing a free donor DNA with homology arms of approximately 1 kbp, it was feasible to successfully perform site-specific insertion of 3 to 4 kbp transgenes. Furthermore, when using the LIG3:4 meganuclease or CRISPR/Cas9 to direct DNA cleavage at site-specific positions, the recombination-mediated integration rates of these transgenes were 0.7% and 2.5 to 4%, respectively. Therefore, it was shown that the frequency of this integration depends mainly on the efficiency of target site cleavage by these endonucleases and, especially, on the efficiency of the HDR pathway, which can be increased in different ways, such as by the type of transgene delivery system in plant cells.

In this sense, Gao et al. (2020b) identified four potential loci in maize chromosomes 1, 3, and 10 for SSTI based on four main criteria: (*i*) regions with conserved haplotype within both non-stiff stalk and stiff stalk germplasm pools, (*ii*) regions with low gene density that are not used in maize breeding, (*iii*) regions with high recombination frequency to minimize donor sequences around the complex trait locus during introgression of the region, and (*iv*) regions that already harbor existing commercially significant agronomic traits. Furthermore, within these four loci, several subpositions were mapped as specific target sequences for CRISPR/Cas9 HDR-mediated insertion, based on the following criteria: (*i*) the target site is at least 2 kbp away from any known gene, (*ii*) the CRISPR/Cas9 target sites are unique in the genome and conserved among the target inbred lines; (*iii*) the 200–500 bp genomic sequences flanking the CRISPR/Cas9 target sites are unique in the genome; and (*iv*) spacing of the CRISPR/Cas9 target sites within a complex trait locus would accommodate genetic crossover to recombine traits. Subsequently, these authors performed site-specific insertion of a landing pad containing heterologous FRT pair sites into previously identified maize loci using CRISPR/Cas9 HDR and then conducted the site-specific insertion of a second desirable transgene within the landing pad mediated by the Flp-RMCE strategy. Transgene insertions into these different CRISPR/Cas9 target sites exhibited a range of transgene expression levels, influencing the expression of neighboring genes to varying degrees, with a transformation rate ranging from 0 to 18% for the landing pad and a Flp-RMCE rate of 38 to 58%. Although *A. tumefaciens*-mediated T-DNA delivery was effective, it showed lower efficiency compared to biolistic-mediated DNA delivery since a larger amount of free donor DNA fragments can be delivered using the biolistics compared to the type IV secretion system (T4SS) (Basso et al., 2020).

Subsequently, Peterson et al. (2021) performed site-specific insertion of three distinct transgenes using CRISPR/Cas9 HDR at target site number 45 (TS45, position 15,177,228 of chromosome 1 based on B73v3 reference genome; Patent number: WO2016040030A1, EP3191595B1), previously identified by Gao et al. (2020b). These authors showed an integration rate of transgenes with right and left homology arms ranging from 8.3 to 10.3% when using the *A. tumefaciens*-mediated T-DNA delivery enhanced with a ternary vector and self-release of donor DNA by Cas9. However, several integration events showed a high attrition rate from T_0_ to T_1_ generation, ranging from 67 to 77%. Similarly, Barone et al. (2020) also performed SSTI in the maize TS45 locus using CRISPR/Cas9 HDR with heat shock-inducible Cas9 expression. Heat-inducible Cas9 simultaneously leads to the release of donor DNA from T-DNA and site-specific cleavage of genomic DNA at the TS45 position at a predetermined time, as immature embryos treated with one heat shock at 45 °C at 4–5 and 11–12 days after inoculation with *A. tumefaciens*. By employing this strategy, these authors obtained an SSTI rate of transgenes with homology arms ranging from 2.7 to 4.7%, lower compared to the constitutively inducible Cas9, but a lower number of chimeric T_0_ plants was observed. It is worth noting that this CRISPR/Cas9-mediated SSTI strategy has been successful in different plant species, such as rice (Sun et al., 2016; Li et al., 2018a; Xu et al., 2020; Dong et al., 2020; Nguyen et al., 2025), tomato (Yu et al., 2017; Danilo et al., 2019), soybean (Li et al., 2015), wheat (Gil-Humanes et al., 2017), and *A. thaliana* (Miki et al., 2018; Wolter et al., 2018), with recombination efficiency rates slightly varying from those obtained in maize. Therefore, the efficiency of SSTI through CRISPR/Cas9 HDR is variable in terms of the plant species, tissue as recipient material, donor DNA delivery system (T-DNA via *A. tumefaciens* and minimal expression cassette via biolistics or viral vector), regeneration of transgenic plants (organogenesis, direct or indirect somatic embryogenesis) and even the size of the transgenes (transcriptional units flanked by homology arms) and the effective size of the right and left homology arms.

Furthermore, *in vivo* CRISPR/Cas9 genome editing of doubled haploids from maternal embryos is feasible in maize, which does not require tissue culture and the use of a chemical chromosome doubling agent, but its application for SSTI remains to be established in maize (Ye et al., 2024). Finally, the simultaneous down-regulation or overexpression of players involved in plant DNA repair machinery, such as the NHEJ pathway, may increase the HDR frequency, but this remains to be established in maize and may lead to other genomic integrity issues and pleiotropic effects (Qi et al., 2013; Chen et al., 2022; Movahedi et al., 2022; Merker et al., 2024).

## 6 CRISPR variants for SSTI

Other endonucleases associated with the CRISPR system, such as Cas12a (Cpf1), are viable for targeting DNA double-strand breaks and SSTI, but HDR efficiency still demands the same requirements outlined for CRISPR/Cas9 and awaits experimental validation in maize (Begemann et al., 2017; Kim et al., 2017; Li et al., 2018b; Lee et al., 2019; Vu et al., 2020). Also, promising results were achieved using CRISPR/Cas9 ribonucleoprotein complexes and single-stranded DNA (ssDNA) as a repair template delivered by biolistics to generate non-synonymous mutations in genes of interest, but this approach also awaits advances in targeting the site-specific insertion of co-delivered transgenes (Svitashev et al., 2016). The Rep-bridged knock-in (RBKI) system, based on the fusion of Cas9 and a viral replication protein (Rep) that acts as a molecular bridge for the *in vivo* enrichment of donor DNA provided as viral replicons and for anchoring the donor DNA at the target site, has been shown as an effective strategy to increase the HDR frequency in rice (Zhou et al., 2025). Similarly, the fusion of Cas9 or Cas12a with rice *Pong* DNA transposases (ORF1 and ORF2::Cas9) and the delivery of a donor DNA within an *mPing* transposon backbone has been established in *A. thaliana* and soybean for SSTI (transposase-assisted target-site integration, TATSI system), providing higher efficiency than that obtained with previously described systems based on recombinases or endonucleases (Liu et al., 2024b; Liang and Wang, 2024). However, the TATSI system still requires improvements to avoid the insertion or transposition of these transgenes of interest outside the planned genomic position, especially caused by the activity of endogenous transposable elements (**Table 1**). These off-target insertion events are even more critical in maize, due to its high content of native transposable elements in the genome. Computational structural biology applied to protein modeling may allow the generation of improved transposase enzymes with increased specificity for new recognition and binding sites, reducing these off-target insertions (Wang et al., 2025b; Ivančić et al., 2025). Furthermore, the TATSI system also introduces over 400 nucleotides from the transposon backbone, along with the sequence of interest (Liu et al., 2024b). Similarly, PrimeRoot editors were successfully used for the precise integration of 4.9 kbp DNA sequences in rice with an efficiency of 6.3% (Sun et al., 2024). In particular, PrimeRoot editors use Cas9 nickase (nCas9) fused to reverse transcriptase (RdDP), two prime editing gRNAs (pegRNAs), and a tyrosine recombinase to integrate ~40 bp recombination sites at a site-specific position determined by the pegRNAs. Subsequently, the recombinase triggers DNA recombination, without DSBs, of transgenes provided by a donor DNA fragment that also contains the respective recombination sites (Sun et al., 2024). PrimeRoot editors are an advancement of twin prime editing (twinPE) and programmable addition via site-specific targeting elements (PASTE), which use an nCas9 fused to RdDP, two pegRNAs, and a serine integrase to direct site-specific insertion of transgenes of >5 kbp (Anzalone et al., 2022) and 36 kbp (Yarnall et al., 2023) in human cell lines. Supporting this, the extended prime editor (EXPERT) and CRISPR-associated transposases (CASTs) have been established in human systems to expand the efficiency of prime editing and the reach of large fragment edits, and improve precise cut-and-paste DNA insertion, respectively (Tou et al., 2023; Xiong et al., 2025).

However, RdDPs exhibit lower fidelity, processivity, and dNTP affinity than many DNA-dependent DNA polymerases (DdDPs) (Liu et al., 2024a). Furthermore, prime editing has the limitation of inserting short DNA fragments, of approximately 40–60 bp, through their pegRNAs (Anzalone et al., 2019; Sun et al., 2024). In this sense, Liu et al. (2024a) developed the DNA polymerase-mediated genome editing (DPET) system, which integrates nCas9 and sgRNA to generate site-specific ssDNA breaks, and the Phi29 fused to the MS2 coat protein (MCP) and a donor DNA-containing template fused at 3’ with MS2 aptamer to direct site-specific insertions of DNA longer than 100 bp, such as two LoxP sites. A similar strategy, named dCas9-SSAP editor, uses deactivated Cas9 (dCas9), gRNA fused at 3’ with MS2 aptamer, and MCP fused to single-strand annealing protein (SSAP, a bacteriophage recombinase that performs precise DNA recombination) to direct SSTI without DSB (Wang et al., 2022a). In the dCas9-SSAP editor, the dCas9 and gRNA::MS2 unwind the dsDNA and recruit the MCP::SSAP to the site-specific position into the genome, when a donor DNA containing homology arms is inserted by recombination mediated by SSAP with an efficiency of up to 20% and low on-target errors and minimal off-target effects in mammalian cells (Wang et al., 2022a). Another strategy that relies on the recruitment of donor DNA to the DSB was named CRISPR-aided targeted T-DNA integration (CRISTTIN), which is based on fusing Cas9 with the 53-amino acid VirD2-interacting motif in *A. thaliana* importin α-3 (IMPα), so that the expression of Cas9 fused to the IMPα (which acts as an adapter), the VirD2 protein (responsible for interacting with the donor DNA or T-DNA), and delivery of the donor DNA as a T-DNA result in a Cas9::IMPα/VirD2/T-DNA bridge or heterocomplex near the DSB (Lin et al., 2025). CRISTTIN is an advancement of the strategy that used the fusion of Cas9 with VirD2 in an attempt to increase HDR efficiency (Ali et al., 2020), proving to be more effective for SSTI than Cas9::VirD2 (Lin et al., 2025). Similarly, retron-based CRISPR/Cas9, nCas9 (D10A), or Cas12a have been established to circumvent the need for a DSB and, primarily, to bypass the need for exogenous delivery of a larger amount of free donor DNA fragments (Khan et al., 2025; Buffington et al., 2025). This retron-based genome editing system allows for a significant increase in the *in situ* production of engineered ssDNA (free donor ssDNA fragment with short homology arms), thus increasing HDR efficiency compared to other systems with exogenous delivery (Jiang et al., 2022). Although efficient for homology-mediated base repair, which accepts short fragments of donor ssDNA as a repair template, recombination-mediated insertion of long dsDNA transgenes remains a limitation of this system for maize, due to its ability to generate in situ only ssDNAs of up to 200 nucleotides (Buffington et al., 2025). In conclusion, several strategies based on CRISPR/Cas9 variants are very promising for implementation in plants, but still require considerable improvements (TATSI) to minor adjustments (PrimeRoot, prime editing, DPET, SSAP, and CRISTTIN) for SSTI in maize. Nevertheless, prior know-how in genome editing of maize can contribute to the successful implementation of these strategies for SSTI in this crop.

## 7 New stable loci for SSTI

Stable loci, or genomic safe harbors, are specific regions within a genome that enable the integration of new genetic material, ensuring functional predictability over multiple generations while preventing adverse effects on the plant phenotype (Dong and Ronald, 2021; Leitão et al., 2025). This concept arises from the recognized influence of neighboring genomic regions on gene expression in eukaryotes, as well as the variation in transgene expression levels observed among transgenic events using the same DNA construct (Dong and Ronald, 2021; Betts et al., 2019). This suggests that the genomic insertion site plays a crucial role in transgene expression levels and should be considered during the development of transgenic commercial varieties or even for functional validation of target genes. In commercial biotechnology programs, numerous transgenic events are produced, genotyped, and phenotyped to identify the most successful event that shows an expression level suitable for effective function of the target trait. This unique event exhibits stable expression across generations, making it suitable for introgression during product development. In this sense, Gao et al. (2020b) selected four chromosomal regions on three maize chromosomes as candidates for stable SSTI, which can be viewed as genomic safe harbors. These regions serve as complex trait loci to facilitate trait stacking. In a complex trait locus, multiple transgenes are positioned and are closely genetically linked, but separated by 50 kbp to ≥1 Mbp (Gao et al., 2020b). This distance is necessary to reduce potential interactions between transgenes and allows the removal of old traits and the addition of new traits through genetic recombination (Chilcoat et al., 2017). Complex trait loci can move as a single genetic locus during introgression into elite lines, decreasing linkage drag and reducing negative impact on plant performance.

There are only a few studies that characterize transgenic insertion sites in maize, and even fewer that correlate these sites with expression levels and transgene stability (Betts et al., 2019; Neelakandan et al., 2023). Neelakandan et al. (2023) characterized 89 insertion sites in 81 transgenic events generated by *A. tumefaciens*-mediated transformation. Insertions spanned all chromosomes, with low correlation between chromosome size and the number of insertions per chromosome. Most insertions were found at the distal ends of chromosomes, in gene-rich regions (64%), indicating that transgenes could potentially influence the expression of neighboring endogenous genes. However, the authors did not characterize gene or protein expression levels and phenotypes of these transgenic events.

The pursuit of genomic safe harbors, particularly for direct SSTI, can be laborious and expensive, and is only justified if the genomic location has a proven strong effect on transgene expression and agronomic performance. Until recently, several academic studies indicated a significant genomic position effect in many species (Dong and Ronald, 2021; Butaye et al., 2004). However, a recent study by Betts et al. (2019) shows that the location of genomic insertion has only a small effect on gene expression levels and agronomic performance when comparing 68 well-characterized maize single-copy transgenic events. These authors argue that confounding factors such as multicopy insertions, unconfirmed intactness, and a lack of biological replications may lead to an overestimation of the position effect observed in previous studies. Betts et al. (2019) examined not only the transgene insertion site but also factors such as *cis*-acting regulatory elements, adjacent transgenes, genetic background, and zygosity, as well as stable expression across generations and agronomic performance in multi-environment trials. Considering all these variables, the promoter and the presence or absence of neighboring transgenes had the strongest impact on transgene expression. Remarkably, most insertion sites of transgenes are agronomically neutral, with no differences in transgene expression observed over three generations. An important finding was the significant effect of genetic background on transgene expression, which can have important consequences for the development of transgenic varieties, where only the most successful event is used for trait introgression during this process. Despite being a robust and well-conducted study, the events used by Betts et al. (2019) underwent an intensive selection process during the tissue culture and molecular characterization phases. Furthermore, the authors did not indicate the insertion sites of the examined transgenes or specify whether these insertions were randomly distributed throughout the genome or more clustered, as highlighted by Neelakandan et al. (2023). Providing this information would help identify potential locations for stable loci used in SSTI.

## 8 Binary and ternary vectors

Constitutive expression of the maize *Baby Boom* (*ZmBBM*) and *Wuschel2* (*ZmWus2*) morphogenic genes has become indispensable for inducing somatic embryogenesis, improving plant regeneration, and increasing the frequency of maize transformation and genome editing (Lowe et al., 2016; Youngstrom et al., 2025). ZmWus2 and ZmBBM are transcription factors that act to increase the production of totipotent cells and drive the development of somatic embryos, respectively (Jones et al., 2019; McFarland et al., 2023). However, their constitutive overexpression is only beneficial during the period between co-inoculation and induction of somatic embryos, as it can cause severe pleiotropic effects in mature somatic embryos and regenerated transgenic plants (Aesaert et al., 2022). Therefore, it is essential to use vector strategies that allow the removal of these morphogenic genes during the co-culture and resting stages of inoculated immature embryos or a system where the morphogenic genes are transiently expressed (without integration into the maize genome) (Hoerster et al., 2020). A successfully used strategy involves the development of T-DNA with expression units flanked by wild-type LoxP pair sites in sense-sense orientation, along with concomitant expression of the CRE recombinase (Aesaert et al., 2022). Delivering the T-DNA or minimal expression cassette into the maize cells leads to transcription of the *CRE* gene and accumulation of CRE enzyme, which recognizes the LoxP sites and removes by recombination the unit containing the morphogenic genes (Hernandes-Lopes et al., 2023). However, morphogenic genes continue to be transiently expressed, although still with a lower probability of integration into the maize genome, while the remaining T-DNA without the morphogenic genes becomes smaller, transformation efficiency increases, and pleiotropic effects are prevented. An alternative to morphogenic genes has been successfully tested through the stable overexpression of Growth Regulating Factors (GRFs), GRF-Interacting Factors (GIF), and GRF-GIF chimeras, which are meristematic and pluripotent competence-associated transcription factors that enhance maize transformation and regeneration without producing pleiotropic phenotypes (Kong et al., 2020; Vandeputte et al., 2024; Hayta et al., 2025). Furthermore, the wound signaling peptide REF1 enhanced plant regeneration in both monocots and dicots, such as stable overexpression of the *ZmREF1* gene increased maize regeneration and transformation efficiency (Yang et al., 2024). Also, *heat shock-complementing factor 1* (*ZmHSCF1*, an AP2 transcription factor) gene overexpression increased the frequency of callus initiation, expansion in the middle area of ​​the callus, and genetic transformation frequency in recalcitrant maize lines (Li et al., 2025). Similarly, the Wuschel‐like homeobox 2a (Wox2a) also induces embryogenic callus proliferation and somatic embryogenesis in maize, without pleiotropic effects in the *Wox2a*‐overexpressing lines (McFarland et al., 2023). Therefore, these three alternatives for morphogenic genes have reduced or even absent pleiotropic effects in transgenic maize that overexpress them, and their adoption may lead to new vector strategies, such as the absence of the Cre/LoxP system, reducing the size of binary vectors.

Due to this complexity associated with morphogenic genes, the size of T-DNA and binary vectors for genetic transformation and genome editing in maize is approximately 30 kbp. Therefore, the manipulation of these vectors for assembly, reconstruction, passage in *E. coli*, and routine use in *A. tumefaciens* represents a considerable challenge. To overcome this, the combined use of multiple binary vectors, each containing a part of the necessary apparatus and delivering minimal expression cassettes through the biolistics, has proven to be a reasonable alternative (Barone et al., 2020; Gao et al., 2020b). In some cases, using optimized binary vectors containing all their units in a single T-DNA is more effective for genetic transformation and genome editing in maize, especially when combined with ternary vectors (Zhang et al., 2019; Peterson et al., 2021).

**Figure 4:**
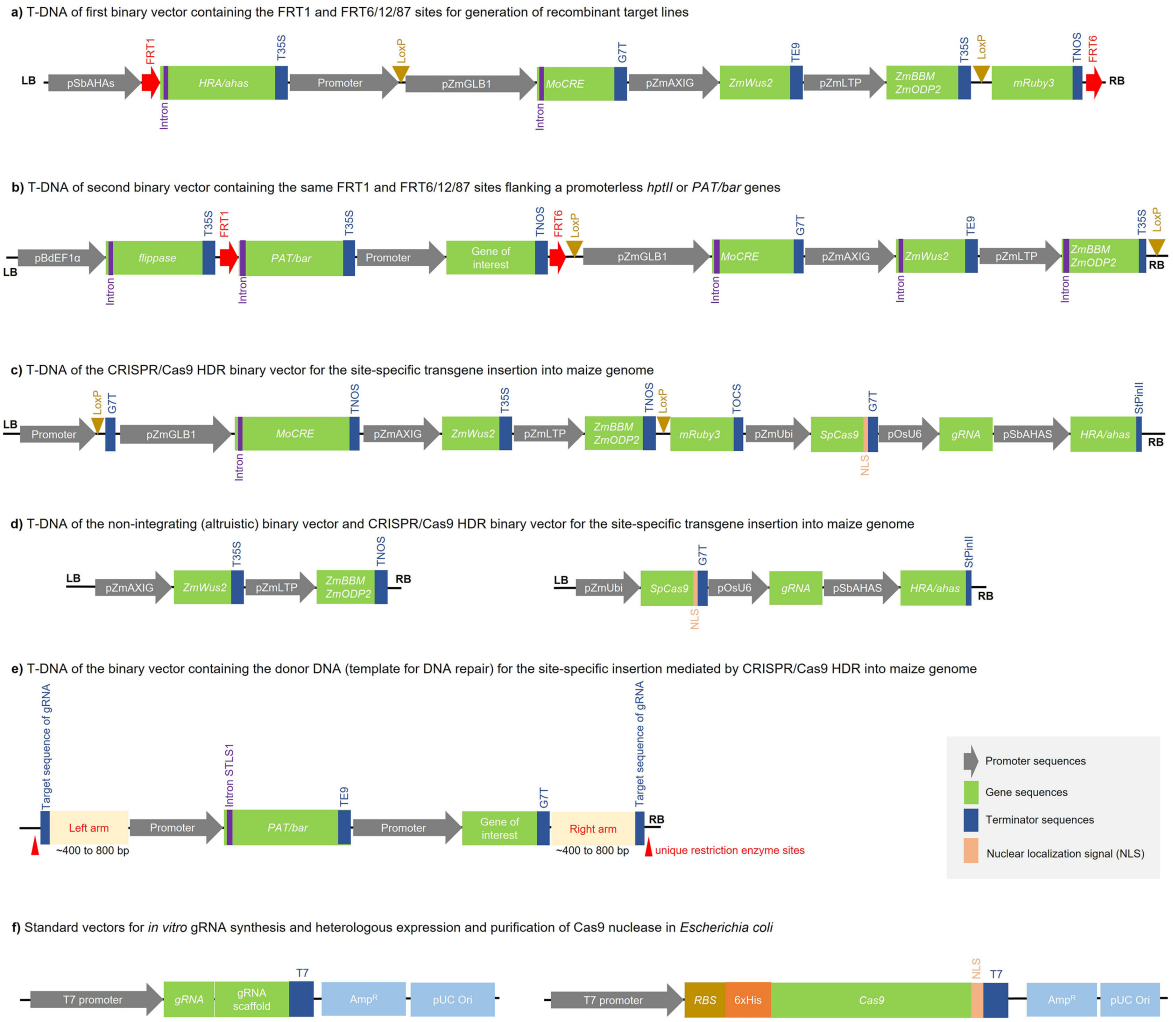
Binary vector and donor DNA strategies for site-specific transgene insertion (SSTI) into maize genome. **(a)** Receptor T-DNA (landing pad) containing FRT pair sites for the generation of recombinant target lines. This landing pad must be randomly inserted into the maize genome. **(b)** Secondary or donor T-DNA containing FRT pair sites for SSTI using the FLP/FRT system in maize. This binary vector containing the donor DNA must be co-transformed into these selected recombinant target lines. **(c)** T-DNA sequence containing genetic elements for site-specific double-strand break (DSB) mediated by CRISPR/Cas9 in maize. The CRISPR/Cas9 vector must be delivered together with the donor DNA fragment (T-DNA or minimal expression cassette) for SSTI. **(d)** T-DNA sequence containing the morphogenic genes in a non-integrating binary vector and T-DNA sequence for site-specific DSB mediated by CRISPR/Cas9 in maize using an altruistic vector strategy. Altruistic vectors must be delivered together with the donor DNA fragment (T-DNA or minimal expression cassette) for SSTI. **(e)** T-DNA sequence containing the donor DNA fragment (desirable transgene flanked by homology arms and target sequence of guide RNA (gRNA) expressed by the CRISPR/Cas9 vector) for SSTI mediated by homology-directed repair (HDR) in maize. The minimal expression cassette must also be flanked by unique restriction enzyme sites to produce the free donor DNA fragment, subsequently delivered onto immature embryos or leaf tissue of maize by biolistics. **(f)** Standard vectors for cloning and *in vitro* synthesis of gRNAs using a commercial kit for *in vitro* transcription and heterologous expression of Cas9 in *Escherichia coli* BL21(DE3) and protein purification by affinity to the 6xHis tag. Then, the purified gRNA and Cas9 protein are assembled to form a ribonucleoprotein complex and delivered together with free donor DNA fragment to maize tissues (immature embryos or leaf) by biolistics. SSTI events are selected *in vitro* based on the selection marker gene present in the donor DNA fragment (*e.g.*, *PAT/bar*). The size of genetic elements within binary vectors is merely illustrative. LB, left border; RB, right border.

Binary vectors containing the landing pad or donor transgenes to direct SSTI through Flp-RMCE are among the vectors with maximum acceptable size, ranging from 25 to 30 kbp (**Figures 4a, b**). In contrast, binary vectors designed to facilitate SSTI through CRISPR/Cas9 HDR exceed this maximum size, ranging from 35 to 45 kbp. Consequently, the T-DNA for CRISPR/Cas9 HDR must be combined in at least two binary vectors. The first contains the machinery to direct site-specific DNA cleavage at the desired position to direct transgene insertion (**Figure 4c**), and the second binary vector containing the donor DNA fragment, which serves as a template for the repair and integration of the desired transgene (**Figure 4e**). Therefore, due to the large size and use of multiple combined binary vectors, the efficiency of maize genetic transformation, genome editing, and SSTI is often reduced. The altruistic transformation strategy or viral-mediated delivery, which delivers morphogenic genes in binary vectors or viral infectious clones separate from the desired transgene for integration, allows for the non-integration of these morphogenic genes, thereby increasing the efficiency of transformation and regeneration of maize with the smaller desirable T-DNA and no morphogenic genes integrated into the genome (**Figure 4d**) (Hoerster et al., 2020; Butler et al., 2025). Ternary vector systems, which encompass an additional accessory, the plasmid carrying *vir* genes, have been used to enhance the T4SS and improve the efficiency of T-DNA delivery and, consequently, facilitate genetic transformation and SSTI in maize (Anand et al., 2018; Kang et al., 2022; Jeong et al., 2025). Importantly, when combining binary and ternary vectors, the origin of replication and antibiotic resistance genes in each plasmid needs to be critically considered for *A. tumefaciens*, ensuring that all these combined vectors maintain and maximize their activity during co-inoculation (De Saeger et al., 2021). On the other hand, the delivery of these minimal expression cassettes through biolistics has served as an alternative to successfully circumvent these difficulties, but this approach can lead to DSBs and undesirable integration, especially very large T-DNAs (Gao et al., 2020b). In this context, the biolistic-mediated delivery of donor DNA fragment in free form (minimal expression cassette) or its self-release from T-DNA through the gRNA/Cas9 itself, by flanking the donor DNA with gRNA target and PAM sequences, has been shown to increase HDR efficiency (Peterson et al., 2021). In this same sense, the *in vitro* synthesis of gRNAs using commercial kits, the heterologous production of Cas9 (**Figure 4f**), their assembly as ribonucleoprotein, and the delivery of these gRNA/Cas9 complexes together with the T-DNA containing morphogenic genes (**Figure 4d**) and the free donor DNA fragment (**Figure 4e**) through biolistics is also a feasible strategy for SSTI, allowing to simplify the vector strategy, but still needs to be optimized for this purpose in maize.

Furthermore, the construction of binary vectors typically requires multiple cloning strategies based on restriction enzymes, Gateway, Golden Gate, Golden Gibson, CasCADE modular vector system, *in vitro* chemical synthesis of large DNA fragments, and cloning in successive stages into entry and/or destination vectors (Hoffie, 2022; Lee et al., 2023). Fortunately, several ternary vectors with diverse origins of replication and different antibiotic resistances are available in non-profit plasmid repositories (Zhang et al., 2019; Aliu et al., 2024). The chemical synthesis of larger DNA fragments and the preparation of customized vectors allow for accelerating the optimization of strategies, validation of genetic elements and genomic insertion sites, and the development of elite events. Designing these binary vectors in anticipation of their eventual use in novel constructs or delivery through biolistics makes prior planning of the expression units essential to allow their exchange or release by restriction enzymes to produce the minimum expression cassette. Therefore, the planned and appropriate use of constitutive, induced, or tissue-specific promoters, terminators, introns, kozak sequences, codon-usage optimization to monocots or maize, the correct orientation of genetic elements, the suitable design of 400–800 bp homology arms, and the selection of gRNAs are fundamental for the proper functioning of Flp-RMCE and HDR strategies in maize (Svitashev et al., 2015; Basso et al., 2020).

## 9 Transgene delivery systems

T-DNA delivery onto immature embryos aiming at SSTI has been mediated mainly by the T4SS of *A. tumefaciens*. In particular, the T4SS is a complex mechanism composed of the VirB and VirD operons encoding at least 16 proteins, which act from the formation of the secretory channel and the extracellular pilus to the transport of T-DNA and related proteins from *A. tumefaciens* to a target plant cell (De Saeger et al., 2021; Jeong et al., 2025). In addition, T4SS allows delivery of T-DNA in a more intact form (lower risk of DSBs) and in smaller quantities for insertion of low copy numbers, usually resulting in insertion of a single copy of the transgene (De Saeger et al., 2021). The size of these T-DNAs and the combination of binary vectors may significantly reduce their delivery efficiency by *A. tumefaciens* (Zhi et al., 2015). Different *A. tumefaciens* strains have been used because they have different performances and characteristics, and may be more suitable for the combination of binary and ternary vectors or genetic strategies. To improve T4SS, in addition to the combination of ternary and binary vectors, preconditioning of *A. tumefaciens* by culturing in a low-salt and low-nutrient medium (usually *Agrobacterium* (AB) minimal medium for 4–16 hours with shaking at 100–150 rpm at 22 °C in the dark) supplemented with 100-150 μM acetosyringone can be used before the co-cultivation phase of bacteria with maize explants (Basso et al., 2017; Kang et al., 2022). T4SS-mediated delivery of T-DNA for random insertion or CRISPR/Cas9 machinery has been satisfactory in different maize tissues (Anand et al., 2019; Wang et al., 2023), but delivery of T-DNA containing the donor DNA fragment for HDR is insufficient for high HDR efficiency. In turn, the T-DNA delivery system by biolistics has been successfully established in the genetic transformation of different crops, including maize (Rech et al., 2008; Wang and Frame, 2009; Raji et al., 2018). Biolistics allows the delivery of a large amount of minimal expression cassette and the transformation of tissues with high recalcitrance, but breakage of this DNA and the random insertion of multiple copies into the plant genome are frequent and undesirable (Dong and Ronald, 2021; Thorpe et al., 2025). However, biolistics has been a viable alternative for the delivery of free donor DNA fragments to increase HDR efficiency in maize (Svitashev et al., 2015; Gao et al., 2020b; Kaul et al., 2024). Furthermore, adjusting the amount of highly pure DNA per bombardment (shot) and delivering free donor DNA fragments (without backbone or extra edges) reduces the risk of breakage and the number of unwanted random insertions. Therefore, the combination of delivery of the T-DNA containing the CRISPR/Cas9 machinery (**Figure 4c**) through T4SS and the free donor DNA fragment (**Figure 4e**) via biolistics can significantly increase the HDR efficiency. On the other hand, transgene delivery through viral vectors (virus-induced genome editing, VIGE) is feasible in maize but still needs to be established for SSTI (Hu et al., 2019; Beernink and Whitham, 2023). Furthermore, viral-mediated donor DNA delivery can amplify the copy number of the repair template, but engineering viral vectors containing long fragments of donor DNA in their genome is a major challenge (Neubauer et al., 2025; Zhou et al., 2025). Similarly, the *in planta* transformation methods of maize with minimal or no tissue culture steps may require special systems or optimized protocols for the delivery of ribonucleoproteins or DNA (Belaffif et al., 2025).

## 10 Maize tissue culture for SSTI

**Figure 5:**
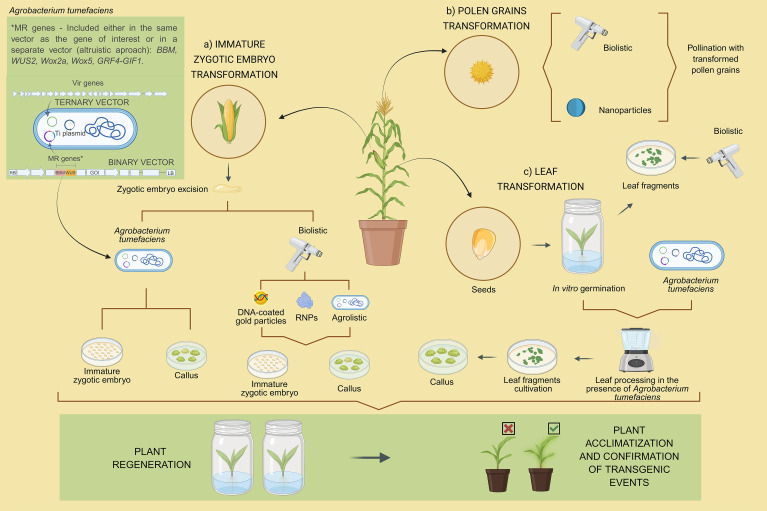
Overview of genetic transformation of maize for site-specific transgene insertion from **(a)** immature embryos collected from fertilized ears under greenhouse conditions, **(b)** from pollen grains collected from plants under greenhouse conditions, and **(c)** from immature leaf tissue derived from the top stalk or leaf whorl collected from very young maize plants grown under *in vitro* tissue culture conditions. The genetic transformation from immature embryos and immature leaf tissue is performed in tissue culture, while the pollen grain-based method is performed under *in vivo* conditions (tissue culture-free strategy). The DNA of interest (T-DNA for transgenesis or donor DNA for homology-directed repair) is delivered into the plant cell through one or two binary vectors mediated by the type 4 secretion system (T4SS) of *Agrobacterium tumefaciens*, with the T4SS being enhanced by a ternary vector. The free donor DNA fragment can also be preferably delivered through biolistics directly onto immature embryos or leaf tissue, combining the T4SS and biolistics. Through biolistics or nanoparticle-based methods, ribonucleoproteins (RNPs) can also be delivered directly into plant tissues. In particular, these two methods of genetic transformation of maize, from immature embryos or leaf tissue, require binary vectors with their genetic elements, culture media, hormones, handling, and *in vitro* culture conditions specifically optimized for each one. MR genes, morphogenic genes, included in the same vector as the gene of interest or in a separate vector in the altruistic approach. Vir genes, virulence genes.

Genetic transformation of maize for random insertion of transgenes, insertion of transgenes containing the CRISPR machinery for genome editing via non-homologous end joining (NHEJ) or HDR, or SSTI through Flp-RMCE or CRISPR/Cas9 HDR has been achieved primarily through the transformation of immature embryos collected from fertilized ears of plants maintained under controlled system in a greenhouse (Yassitepe et al., 2021; Hernandes-Lopes et al., 2023). Although the transformability of maize immature embryos mediated by *A. tumefaciens* for transgenesis or genome editing is highly genotype-dependent, with the Hi-II A × Hi-II B progeny (Svitashev et al., 2015, 2016; Cody et al., 2020) and the B104 inbred line (Kang et al., 2022; Aesaert et al., 2022; Lee et al., 2023; Hernandes-Lopes et al., 2023) being the most amenable and accordingly common choices, advances in tissue culture and morphogenic genes have significantly broadened the range of transformable genotypes (Lowe et al., 2018; Kausch et al., 2021; McFarland et al., 2023; Hernandes-Lopes et al., 2023). Consequently, increasing the ability to transfer or introgress the transgene or edited alleles obtained in these donor lines into commercial elite cultivars (recipient lines). Furthermore, other genotypes have been used less frequently for genetic transformation of maize, such as PH184C, HC69, PHH5G (Gao et al., 2020b), HC69, PH2RT (Anand et al., 2019), and CML360, CML444, and PCL1 (Hernandes-Lopes et al., 2023), while B73 is among the most difficult inbred lines to obtain transgenic events (Mookkan et al., 2018). However, the significant variation that exists from ear to ear greatly limits the efficiency and uniformity of genetic transformation rounds of maize. Furthermore, the production of ears with a considerable number of grains, containing highly viable and uniform immature embryos for use in tissue culture, is still not trivial and is limited to certain seasons (Ishida et al., 2007; Hernandes-Lopes et al., 2023). These limitations are imposed by environmental conditions such as temperature, photoperiod, and light intensity and quality. To ensure successful plant transformation and regeneration, donor plants of immature embryos must be grown under strictly controlled nutritional, sanitary, and environmental conditions, requiring highly optimized greenhouse infrastructure (Yassitepe et al., 2021). In recent years, substantial efforts have led to the development of optimized protocols and vectors containing morphogenic genes (Lowe et al., 2016; Masters et al., 2020; Hoerster et al., 2020; Vandeputte et al., 2024) that increase the virulence of *A. tumefaciens* (Anand et al., 2018; Zhang et al., 2019; Kang et al., 2022; Aliu et al., 2024), which significantly improved the efficiency of embryo-dependent transformation and allowed recalcitrant genotypes to be transformed as well (Masters et al., 2020; Hernandes-Lopes et al., 2023) (**Figure 5a**). The identification of hypersusceptibility genes to *A. tumefaciens*-mediated transformation in maize and their subsequent knockout or RNAi-mediated down-regulation may be an alternative to increase the efficiency of genetic transformation, genome editing, and SSTI of these recalcitrant genotypes (Liu et al., 2025; Doorly et al., 2025; Dodds et al., 2025). Also, tissue culture-free methods based on genetic transformation of pollen grains and subsequent maize pollinization are being developed (**Figure 5b**) (Wang et al., 2022b). As another alternative to this limitation imposed by immature embryos, genetic transformation of maize through leaf tissue has been successfully implemented for the random insertion of transgenes (Wang et al., 2023; Azanu et al., 2025; Kumar et al., 2025). This transformation method uses immature leaf tissue derived from the top stalk or leaf whorl collected from very young maize plants germinated *in vitro* (**Figure 5c**). It also requires vectors with morphogenic genes controlled by a removal system and some genetic elements specific to leaf tissue (*e.g.*, promoter sequences), while the *in vitro* selection and regeneration system of transformants requires very particular conditions, such as different types and concentrations of hormones, appropriate culture medium, growth room conditions, and handling (Wang et al., 2023; Kumar et al., 2025). Although not yet fully established in different laboratories for large-scale maize transformation and genome editing, this method may allow greater uniformity of the source explant used in transformation and wide availability of this material throughout the year. Therefore, it is expected that more laboratories will adopt and adapt these leaf-based protocols, given their potentially simpler infrastructure requirements compared to protocols based on immature embryos produced in a greenhouse. Fortunately, both transformation methods through immature embryos and leaf tissue are compatible for genetic transformation of maize with binary vectors containing CRISPR/Cas9 HDR strategies, as well as for delivery of free donor DNA fragments or minimal expression cassette via biolistics (Wang et al., 2023). In particular, the advantage of the method using immature embryos is that it is fully established in different laboratories and it delivers high efficiency of transformation and regeneration of transgenic and/or edited plants, with a reduced number of escapes and chimeric events. Meanwhile, transformation via leaf tissue still requires some advances, optimizations, and establishment in different laboratories, as well as the imminent need to optimize DNA delivery through biolistics.

## 11 Genotyping and phenotyping of transgenic lines with SSTI

RTLs generated by random insertion of landing pads for Flp-RMCE must undergo rigorous molecular characterization after tissue culture and throughout subsequent generations. This characterization is essential to ensure the integrity and stability of the gene insertion, employing molecular approaches such as PCR, Sanger sequencing, real-time RT-PCR, Southern blot, genome walking, RNA-Seq, and high-throughput genomic sequencing (Zeng et al., 2020; Neelakandan et al., 2023). This selection should prioritize RTLs that harbor a single intact copy of the transgene inserted into neutral genomic regions, such as regions that do not interfere with neighboring genes or endogenous genetic elements. Additionally, the identification of homozygous events with high and stable transgene expression is critical to enable subsequent co-transformation using a second donor transgene (Anand et al., 2019). Similarly, transgenic events generated by co-transformation and Flp-RMCE must also be subjected to molecular characterization after tissue culture and over generations to monitor the recombination and stability of the transgene. Elite transgenic lines obtained by Flp-RMCE with SSTI of a single and intact copy of this second transgene, with high and stable expression over generations and in homozygosity, should be prioritized for subsequent phenotypic analysis.

Meanwhile, transgenic events generated by CRISPR/Cas9 HDR containing SSTI must undergo rigorous molecular quality control after tissue culture to ensure the insertion and integrity of this transgene. Furthermore, the advancement of maize generations must be accompanied by molecular analysis to support the assisted segregation of the CRISPR/Cas9 transgene, which must be eliminated to obtain elite transgenic lines with SSTI, but without Cas9 and morphogenic genes. It is common to have low HDR efficiency and high attrition rates in transgenic maize lines generated by this strategy, which are the result of imperfect recombination events (Svitashev et al., 2015; Barone et al., 2020). Therefore, it is necessary to monitor the integrity of the donor transgene inserted at the site-specific position in the T_0_ generation until homozygosity is achieved, using assays such as PCR and Sanger sequencing of the entire transgene and flanking regions. Even knowing in advance that the insertion site may confer greater stability to a transgene of interest, it is still necessary to monitor the stability of this new transgene and neighboring genes over generations through real-time RT-PCR, RNA-Seq, and Western blot. This is because each transgene can contain different genetic elements, structural organizations, and distinct DNA sequences, which can bring instability to the region of its insertion and to itself. Elite transgenic lines obtained by CRISPR/Cas9 HDR with site-specific insertion of a single and intact copy of the donor transgene, with high and stable expression over generations, in homozygosity, and without Cas9 and morphogenic genes, should be prioritized for phenotypic analysis (Peterson et al., 2021). Likewise, transgenic events containing landing pads for Flp-RMCE inserted by CRISPR/Cas9 HDR must undergo the same rigorous quality control described above (Gao et al., 2020b). Further analyses of epigenetic marks in these transgenes inserted into the maize genome must also be constantly addressed to monitor the stability of these traits over generations (Dobránszki et al., 2025). Finally, biosafety regulations for the commercialization of these transgenic lines with SSTI associated with agronomic traits may certainly require more comprehensive molecular analyses to demonstrate that there is no insertion of undesirable genetic elements, off-target effects, or any interference with other native genetic elements.

Phenotyping of these maize transgenic lines generated by any of the three insertion approaches described above begins in the growth room and greenhouse with a reasonable number of homozygous lines that have passed the quality control based on molecular analyses. The best transgenic lines, with superior agronomic performance under these controlled conditions, should also be verified in open-field and cross-stress conditions (Betts et al., 2019; Peterson et al., 2021). For both environments, biochemical and phenotypic analyses appropriate to the agronomic trait, sensitive and accurate, should be used on a case-by-case basis for each transgene inserted into the transgenic line being phenotyped. Desirable elite lines for field trials should not contain apparent penalties in biological processes or agronomic traits resulting from SSTI, but rather an improved or novel agronomic trait conditioned by the transgene. Therefore, the rigor placed on each of these analyses will be decisive for the success of identifying the best transgenic lines for commercial approval (Gao et al., 2020b; Yassitepe et al., 2021).

## 12 Concluding remarks and perspectives

The development of transgenic maize, for the expression of transgenes or molecules of interest, continues to be important to achieve desirable agronomic traits, such as improving plant resistance to insect pests and plant pathogens, and tolerance to new herbicides and abiotic stresses. The development of elite transgenic events through random insertion of transgenes is still costly and laborious. Therefore, precisely targeting the insertion of these transgenes to genomic safe harbors can greatly reduce the effort expended compared to random insertion. RTL lines containing landing pads engineered for recombination mediated by flippase or Cre recombinases are an alternative that allows successful two-step insertion of a transgene of interest at a site-specific position, but still requires considerable effort, time, and financial resources. SSTI in genomic safe harbors in a single step mediated by genome editing tools is a powerful alternative, but requires prior knowledge of these positions. Also, strategies based on meganucleases, ZFNs, and TALENs are complex to engineer and less effective than CRISPR/Cas9 HDR. Although CRISPR/Cas9 stands out as the best alternative, it still has active intellectual property, which may give preference to these other genome editing technologies for the developing commercial products. In this scenario, SSTI mediated by recombination using Flp-RMCE and genome editing systems has important limitations and advantages to be analyzed for each project. The main characteristic of both strategies is the low efficiency of SSTI at the desired position, but it is still considered viable compared to random insertion. Fortunately, important technological advances have been made in recent years that allow for greater RMCE and HDR efficiency. Among these advances, the use of iterative Cre-LoxP, TATSI, EXPERT, CASTs, RBKI, DPET, and CRISTTIN systems, PrimeRoot and dCas9-SSAP editors, and λ-red recombineering and bridge RNAs stand out, but these still need to be established or considerably optimized for maize. Furthermore, identifying these genomic safe harbors for SSTI in maize is a challenge that needs to be overcome, but will be very important for stacking multiple transgenes at the same locus and facilitating the transfer of this complex trait locus during maize breeding.

In laboratory terms, significant advances are still needed to enhance the efficiency of SSTI, encompassing the optimization of genetic elements and binary vectors, DNA delivery, genetic transformation, and plant regeneration, as well as the molecular and phenotypic characterization of elite plants. Therefore, optimizing the maize genetic transformation through leaf tissue, increasing the efficiency and uniformity of transformation through immature embryos, optimizing the genetic elements and binary vectors, optimizing and reducing the cost to synthesize long DNA fragments, combining binary with ternary vectors, improving the delivery system for free donor DNA fragments, identifying new genomic safe harbors, making genotyping by sequencing more cost-effective for a large number of samples, and investing in research and innovation to improve the sensitivity and accuracy of phenotyping assays are necessary advances for the adoption and efficiency of SSTI. Meanwhile, recent genome editing technologies based on ribonucleoprotein, the double haploid *in vivo* genome editing system, novel morphogenic genes that improve maize regeneration without pleiotropic effects, and maize transformation through leaf tissue hold great promise for SSTI in maize. In conclusion, SSTI has been successfully established in maize, with emphasis on the FLP/FRT and CRISPR/Cas9 HDR systems, but important advances remain to be made to increase RMCE and HDR efficiency and support the large-scale commercial application. Therefore, recent technologies, such as those based on prime editing and polymerases, which have been successfully established in human and plant cells, are capable of bringing about significant transformations by substituting the random insertion with site-specific insertion in maize.
